# Prescription pattern in asthma therapy at Gorakhpur hospitals

**DOI:** 10.4103/0970-2113.59261

**Published:** 2010

**Authors:** Awanish Pandey, Poonam Tripathi, Rishabh Dev Pandey

**Affiliations:** Division of Pharmaceutical Sciences, Institute of Technology & Management, GIDA, Gorakhpur, U.P., India

**Keywords:** Prescription-monitoring, out patient department, asthma

## Abstract

**Objectives:**

This prescription-monitoring study was conducted to establish the drug-prescribing trend of anti-asthmatic drugs in various hospitals of Gorakhpur.

**Setting:**

The study covered three famous hospitals of Gorakhpur.

**Patients and Methods:**

Hundred patients were studied using a prescription auditing performa. Data was recorded from the patient's attending the out patient department using a chance random sample method for two months. Patients who co- operated were interviewed and information was filled in the performa.

**Results:**

The results suggested that b-agonist (40%) were the most frequently prescribed anti-asthmatic drugs followed by Methylxanthine (27%), corticosteroids (25%), leukotriene antagonist (4.4%) and anti-histaminics (3.6%) was the least prescribed. Analysis of prescription revealed that multiple drug therapy (81%) was opted for a significant number of patients as compared to single drug therapy (19%). Contrary to popular belief, oral dosage form tablets (56.3%) were preferred over inhalation (33.8%).

**Conclusion:**

It is concluded that the present prescribing pattern of anti-asthmatics in Gorakhpur does not completely meet standard guidelines of asthma treatment. Hence there is a need to encourage physicians of Gorakhpur to follow the guidelines while treating asthma.

## INTRODUCTION

Asthma is a disease of airway inflammation and airflow obstruction characterized by the presence of intermittent symptoms including wheezing chest tightness dyspnea and cough together with bronchial hyper responsiveness. In India three to five per cent pediatric population is affected by asthma whereas in adults the prevalence range from 3-11%.[1] Asthma is a chronic diseases resulting in high mortality and morbidity in today's world.[2] Long-term treatment is generally required for an effective management, which has an effect on the cost of the therapy and patient's compliance.[3]

Drug utilization audits are qualitative assurance programs to ensure that drugs are used correctly and safely. The nature of such audits can be quantitative or qualitative or combination of both. Quantitative audits are concerned with quantifying various facts of drug therapy use within a health care system area group whereas qualitative audits compare drug use or practice with predetermined standards or criteria.[4] The present study aimed to assess drug utilization in asthma therapy as a quantitative type of prescription auditing to generate data with respect to their extent variability of drug usage in a health care system of a particular criteria

## PATIENTS AND METHODS

The study was conducted at various hospitals after obtaining consent to collect information from patients attending the physician in out patient department. One hundred prescriptions from six physicians were collected from randomly selected three hospitals at various places of Gorakhpur for a period of two month (July to August 2008). Individual patients were interviewed using the prepared questionnaire for this study after their visit to the doctor.

The patients who co-operated were interviewed and information was filled in Performa. The tool used was a set of prepared questionnaire for each patient whose diagnosis was based on clinical evidence provided by the doctor and the technique adopted was personal interview with the patient. All the patients were asked for information as specified in the questionnaire. Their habits, socio-economic status and occupation were also asked as mentioned in the patients' information. Verbal consent was taken from every patient before enrolling in this study. This was an observational study aimed at identifying the current practice with anti-asthmatic prescription. Questions were asked about the smoking status and educational level and family history of any bronchial disorder. The number of drugs per prescription was observed. Route of administration of drugs recorded.

### Study variables of data

The study variable in the study are - age, sex, smoking, occupation, family history, clinical diagnosis, anti- asthmatics prescribed, single/multiple drug therapy, brand name, generic name, dosage forms of anti-asthmatics.

Physicians take a medical history to establish the diagnosis of asthma. Identification of symptom patterns, severity of symptoms and precipitating factors will support the diagnosis of asthma: *How often and when do episodes occur?* *What is their duration? Do symptoms occur or worsen during the night, with exercise or with an infection? Are they precipitated or aggravated by specific triggers? Do they interfere with sleep or daily activities, or require emergency department or hospital visits?* Some physicians use pulmonary function test for the confirm diagnosis of asthma. Spirometery or peak expiratory flow performed before and after the patient inhales a short acting bronchodilator to assess airflow obstruction.

### Inclusion and exclusion criteria

Only outpatients suffering from asthma alone were included in the study. Asthmatic patients who suffered from other disease such as hypertension, heart problem and other problems such as peptic ulcer, diabetes mellitus and migraine were excluded from the study. Patients of acute bronchitis, chronic bronchitis and pneumonia were excluded.

### Analysis of data

1. Compilation of data was done.
2. Data were classified in different independent variable.
3. The data was tabulated using Excel in the computer.
4. Percentage was calculated.
5. Using EXCEL-2000 plotted graphs.

## RESULTS

The drugs used primarily in asthmatic patients in Gorakhpur hospitals are presented in Table 1. Demographic analysis of data revealed that the study population comprised of more male than female. Majority of the patients were in the age group of 40-50 years [Table 2]. About two per cent of females and six per cent of the males had a family history of asthma.

**Table 1:** Drugs primarily used in Gorakhpur for asthma

Category	Name of the drugs
Corticosteroids	Beclomethasone, Budesonide
β-agonist	Salbutamol, Terbutaline, Salmeterol
Methylxanthine	Theophylline and Etophylline
Leukotriene antagonist	Montelukast
Anti-histaminics	Loratadine

**Table 2:** Demographic distribution of asthmatic patients

Age groups (yrs)	Male (n = 78)	Female (n = 22)	Total
0-10	3 (3)	1 (1)	4 (4)
10-20	9 (9)	3 (3)	12 (12)
20-30	7 (7)	1 (1)	8 (8)
30-40	12 (12)	5 (5)	17 (17)
40-50	29 (29)	5 (5)	34 (34)
50-60	18 (18)	7 (7)	25 (25)

Value indicated in () are in percentage.

The results of this study showed that most of the patients received multiple drug therapy compared to single drug therapy. In multiple drug therapy, two-drug combinations were as more widely prescribed than combinations of three/four drugs [Table 3].

**Table 3:** Drug therapy regimen (single/multiple drug regimen)

Drug therapy	Number of patients
Single drug	19 (19)
Multiple drug	81 (81)
Two drug	42 (42)
Three drug	29 (29)
Four drug	13 (13)

Value indicated in () are in percentage

The pattern of drug prescription in asthmatics showed the highest prevalence of β-agonist followed by methylxanthine and corticosteroids [Table 4]. Anti-histaminics was the least prescribed anti- asthamatic drug and is mostly given in tablet form followed by inhaler and other formulation like syrup [Table 5].

**Table 4:** Anti-asthmatic drug utilization (individual/combination)

Anti-asthmatic drugs	Therapy		
	
	Individual	Combination	Total
β-agonist	78	12	90 (40)
Methylxanthine	47	15	62 (27)
Corticosteroids	31	25	56 (25)
Leukotriene antagonist	10	-	10 (4.4)
Anti-histaminics	8	-	8 (3.6)

Value indicated in () are in percentage

**Table 5:** Route of administration (dosage form)

Dosage form	Number of prescription
Tablet	80 (56.3)
Inhaler	48 (33.8)
Syrup	14 (9.9)

Value indicated in () are in percentage

## DISCUSSION

A prescription-based survey is considered one of the scientific methods to assess and evaluate the rationality of the prescription. Recommendations of various international bodies on asthma which help improve prescribing practices of the physicians and ultimately clinical standard are now available.[5–7] Analysis of 100 cases of asthmatics, in this study, revealed that asthma was more prevalent in males than females. Demographic characteristics also showed that males (78%) were suffering more from asthma than females (22%). Most physicians diagnose the asthma by history and examination. Drummond *et al*. suggest that improving access to spirometry in primary care may improve accurate diagnosis and compliance with guidelines.[8] However, many GPs, especially those from regional areas, thought spiro-meters too expensive, and some GPs lacked confidence in their use. Our study suggests a significant divergence between recommendations regarding spirometry and GPs' confidence to perform and interpret the tests. Since asthma patients often require more than one drug for control of symptoms hence combination are required to treat asthma. In this study 81% patients were on multiple drug therapy and only 19% patients were on single drug therapy. This indicated the awareness among prescribes. Overall drug utilization showed β agonist (40%) be maximum used category. Anil Kumar *et al*. also showed similar trend.[9] Analysis suggests that symptomatic relief agents are more prescribed than asthma controlling agents. The inhalational route causes a high local delivery therapy hence will improve the therapeutic ratio and minimize systemic side effects. According to treatment guidelines inhalational therapy for asthma should be the first choice but only 30.9% patients, at the time of study, used inhalers. This could be because prescribers do not believe in prescribing it or on the patient's side, the level of acceptance is low apart from non-compliance and coordination associated with use of inhaler. Therefore, it is suggested that physicians prescribe inhalers more than tablets. However, this will require more of patient's education and convincing by the treating physician for inhalational therapy. When antibiotics, expectorants, anti-tussive and anti-histaminics are less prescribed compared to asthma controllers, it suggests awareness among physicians towards the standard treatment guidelines

This study concluded that the present prescribing practice in asthma therapy in Gorakhpur is not sufficiently rational. Based on these base line data and lacunae, in the present prescribing practice, an intervention was suggested to improve the current prescribing trend for better and rational utilization of drugs. There is need to encourage physicians to follow asthma guidelines while managing asthmatic patients. We recommend that these practitioners can be used as facilitators in future training programs for general practitioners, family physicians and primary care physicians. In conclusion, National Asthma Education Program will be beneficial as an initial step, in improving asthma knowledge and increasing awareness in the medical community, on current therapy. This study may be more meaningful to improve, further, the prescribing through successful implementation of interventional program in the health care centers.
